# Making a case for the free exploratory paradigm: animal welfare-friendly assays that enhance heterozygosity and ecological validity

**DOI:** 10.3389/fnbeh.2023.1228478

**Published:** 2023-08-03

**Authors:** Michael H. Parsons, Rafal Stryjek, Markus Fendt, Yasushi Kiyokawa, Piotr Bebas, Daniel T. Blumstein

**Affiliations:** ^1^Department of Biological Sciences, Fordham University, Bronx, NY, United States; ^2^Institute of Psychology, Polish Academy of Sciences, Warsaw, Poland; ^3^Institute for Pharmacology and Toxicology, Center for Behavioral Brain Sciences, Otto-von-Guericke University Magdeburg, Magdeburg, Germany; ^4^Laboratory of Veterinary Ethology, The University of Tokyo, Tokyo, Japan; ^5^Department of Animal Physiology, Institute of Functional Biology and Ecology, Faculty of Biology, University of Warsaw, Warsaw, Poland; ^6^Department of Ecology and Evolutionary Biology, University of California, Los Angeles, Los Angeles, CA, United States

**Keywords:** animal welfare, research animals, rodents, laboratory mice, laboratory rats, behavioral protocols

“*Rodents are not test tubes with whiskers”*

–* author unknown*

## Introduction

Rodents, laboratory rats and mice, have been used as models in experimental research for almost two centuries (Keeler, 1947; Bolles and Woods, 1964; Nishioka, 1995; Guénet and Bonhomme, 2003). During this time, it has been assumed that rodent suffering was a necessary part of the tremendous scientific advancement, and thus the means justified the ends. After centuries of unregulated research, animal welfare committees were instituted in Europe, America and Australasia to limit animal suffering (Steneck, 1997; Curzer et al., 2016). While licenses to conduct research on animals are often burdensome to obtain (Curzer et al., 2016), there has been strong variance across nations in expectations for the license, the review process and compliance (Varga, 2013). Institutions in some nations, for instance, are not financially-equipped to perform random onsite inspections, or hire veterinarians to assess or enforce conditions of the license. Those critical of the process, such as Rollin (2002) have invoked the idiom “*the fox guarding the hen-house”* to describe the seemingly voluntary nature of compliance for researchers in these circumstances. Regardless, some common assays that can cause needless or unjustified suffering are still used (Mason et al., 2004; Carbone, 2019), and some licenses that are appropriately established are not followed closely-enough (Jerusalem Post, 2023). Meanwhile, science is more broadly communicated than ever, and the general public and media are becoming more aware of this suffering, particularly as we learn more about the animals themselves. For instance, rodents were historically viewed as vermin or pests. Yet it is now widely recognized that rodents are sentient (Bartal et al., 2011, 2014; Mogil, 2012; Mason, 2021), and like any animal, they deserve an expansion of our “compassion footprint” (Bekoff, 2010; Cochrane, 2013; Dunayer, 2013).

Amidst challenges to the current system (Varga, 2013) mounting data on animal suffering (Buckland and Nattrass, 2020; Webb et al., 2020), and calls from animal rights groups (McMahon et al., 2012) recommending the replacement of laboratory animals altogether (Gruber and Hartung, 2004; Langley et al., 2007; Robinson et al., 2019), there is a clear need to take additional steps to limit suffering. One such approach is to develop alternative assays. Fortunately, there are available assays which promote more positive affective states for rodents (Jirkof et al., 2019), while minimizing the number of animals bred into captivity and/or euthanized. Here we argue the value of one of many such approaches, the free exploratory paradigm (FEP; Griebel et al., 1993), which is a paradigm that allows animals to freely enter and exit a test apparatus. We suggest that the FEP can improve rodent welfare in both laboratory and field assays. We then discuss how the FEP can be utilized to improve the quality of data from some of these experiments.

As a team composed of field ecologists, ethologists, physiologists and neuroscientists, we study rodents in the field and laboratory. Our experiences with rodents do not align with historical attitudes and opinions. Rodents have traditionally had a reputation, particularly in some nations, as animals that “deserved to die” (Buckland and Nattrass, 2020). This poor perception of rodents was mostly worldwide, but it was epitomized by a survey of 200 households in Cape Town, South Africa. Almost one-fifth of participants answered they were “happy” for rodents to suffer before death, and only one third cared whether rodent control was humane (Buckland and Nattrass, 2020). In the centuries that unregulated rodent research took place, attitudes toward rodents used in scientific research could have reflected social attitudes. Common tests that have historically been known to cause suffering included moderate deprivation and reward studies, forced swim tests and forced copulation assays. In the latter case, sexually-receptive females are first paired with a male and later substituted with non-receptive females. These non-consensual copulatory assays were “justified” by the authors as a means to better understand (human) male sexual violence. Despite the unquestioned importance of laboratory animals to scientific progress over 200 years, suffering has become institutionalized. Not only is suffering bad for welfare, but stress within the laboratory causes data- distortion and reduces the justification of such studies (Bailey, 2018). Fortunately, we are now far enough along in advancements and technology, that we can raise the standard of justification for a few historic assays that have limited usefulness.

For instance, all members of our international team have experience in field research. Several of our team members have been approached by laboratory researchers who wish to expand their studies to the field. The reasons for the transition are varied, yet one experience stands out. When MHP inquired about the forced swim test to a research team who recruited him to help, they explained that the assays were used to train future research students and for the benefit of any theoretical knowledge that was gained by using it. The principal researcher, who worked at a major research institution, had not considered whether there were tangible outcomes to medicine or society. However, some of these researchers-in-training would likely carry the same assays forward when they train their own students. One can see how this attitude, if embodied elsewhere, could become a cyclical process that perpetrates suffering. This occurs when students become desensitized to rodents' suffering (Balcombe, 2000), develop “compassion fatigue” (LaFollette et al., 2020), or falsely assume suffering is justifiable, because “vermin” are not thought of as having “feelings, emotions and/or memories.”

Research over the past 15 years, however, has shown laboratory rodents experience a wide range of feelings, emotions, regret and intelligence—being far more sentient than previously thought (Webb et al., 2020; Crump, 2022; Webster, 2022). While all animals, sentient or not, deserve our compassion (Bekoff, 2010), society has historically given more rights to animals thought to express memories, intelligence or sentience (Cochrane, 2013; Dunayer, 2013). For instance, we now know rats and mice show a high degree of empathy (Crawley, 2004; Bartal et al., 2011, 2014; Cox and Reichel, 2020) and remorse (Steiner and Redish, 2014). Rats are smart (Davis, 1996), have exceptional memories and can assess time (Kononowicz et al., 2022). Among rats driving robotic cars, those living in enriched environments had more robust driving skills (Crawford et al., 2020). All 17 rats of the latter study assayed had a higher concentration of dehydroepiandrosterone while driving, indicating they were experiencing the reward of learning a new skill (Crawford et al., 2020). The media, so important in steering social expectations, also widely-reported rats' ability to play, be tickled, and express joy through ultra-high frequency vocalizations (Mällo et al., 2007; Hammond et al., 2019; Burke et al., 2022). In a highly-cited, and attitude-shifting paper, these “chirps” were shown to be analogous to laughter (Panksepp and Burgdorf, 2000). Finally, in a finding that went “viral” on social media, researchers found that rats move to the beat (“danced”) of an eclectic range of popular music from Mozart to Michael Jackson (Ito et al., 2022). This public knowledge is helping shift attitudes, which is in turn compel animal rights advocates and researchers to explore alternatives. While a common concern of researchers is that their research outcomes could be compromised by welfare-friendly designs, we will argue that, in some situations, the FEP may actually improve data quality and research outcomes.

Ironically, the researchers who we referred to earlier, contacted us, not to improve the welfare of the animals, but to increase the value of their own research. The principal complaint was that “*laboratory animals were a product of indolence and lacked genetic variability.”* To eliminate variation, which might permit smaller effects to be detected, laboratory studies often used genetically-homogenous strains. Not only have laboratory animals been deprived of heterozygosity, the processes of domestication has modified the behavior and physiology of these animals. Such studies make it difficult to have broad conclusions. To eliminate further variation, laboratory studies also test animals in standardized environmental conditions that often do not reflect their natural environments in which they evolved. Thus, traditional tests purposefully remove interfering contextual variables (Rader, 1997, 2004; Würbel, 2000; Wolff, 2003; Voelkl et al., 2020). Yet these environmental variables intentionally removed from standardized assays may be essential for understanding treatment of some illnesses (Nesse, 1994; Mobbs and Kim, 2015; Oppenheim, 2019).

For instance, much neuroscience research focuses on fear or anxiety. These are natural states (Blumstein, 2020) that are elicited by cues of threats and may be modified in the absence of shelter, the presence of conspecifics, or when visibility changes (Orrock and Danielson, 2009; Parsons and Blumstein, 2010). The lack of these sorts of natural contexts in research assays, as well as a lack of genetic variation, has led to a perceived crises in some sub-disciplines when laboratory outcomes do not relate to practice (Manjili, 2013; Drucker, 2016; Fendt et al., 2020; Stryjek et al., 2021a). Furthermore, we understand that non-welfare-friendly designs may create uninterpretable data. This occurs when data is compromised after being collected from stressed animals (e.g., data distortion; Bailey, 2018). Yet the FEP, as we describe below, could improve research outcomes to address each of these crises.

The FEP can be a more welfare-friendly approach when used in the laboratory (Stryjek et al., 2012; Kohl et al., 2018; Mei et al., 2020; Kohler et al., 2022) or the field (Stryjek et al., 2018; Bedoya-Pérez et al., 2021; Parsons et al., 2023). This type of assay is similar in some respects to home cage testing (Grieco et al., 2021), where animals are tested in the place they live in order to minimize the stresses of transport and handling. It also allows animals to choose if and when they visit an experimental test. They are neither deprived nor punished beforehand, and they choose whether to remain or leave a test arena. There are many examples of FEP and we will give only generalized examples of how they might operate.

**Figure 1:**
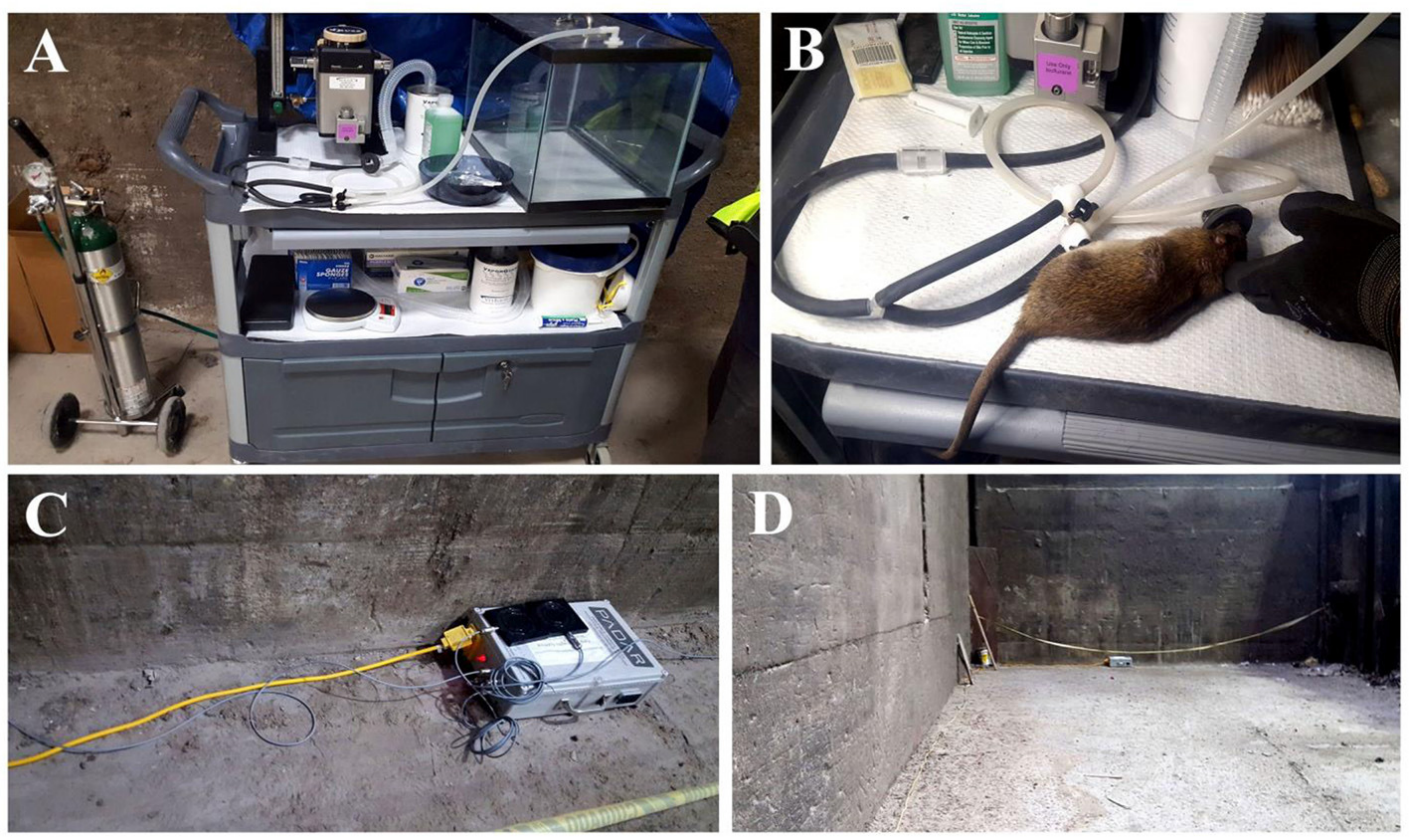
Field FEP comprised of free ranging animals with continuous surveillance using passive microchip readers and video recorders in an urban environment. **(A)** Mobile outdoor laboratory; **(B)** gas anesthesia system for RFID implantation; **(C)** antennas placed within “rat runways”; **(D)** natural (non-modified) landscape within an urban warehouse setting.

For instance, an FEP test may involve experimental chambers whereby animals are attracted by food, shelter or conspecifics, and assayed under video surveillance or direct observation (Bedoya-Pérez et al., 2021; Parsons et al., 2023). In some circumstances, even more realistic assays may be constructed using the natural landscape such as common “rat runways” instead of chambers (Figure 1; Parsons et al., 2019). While we recognize these approaches are not sufficient for all research questions, we will highlight the benefits, a few types of hypotheses that may be addressed, and potential advantages over traditional tests.

### Field

**Figure 2:**
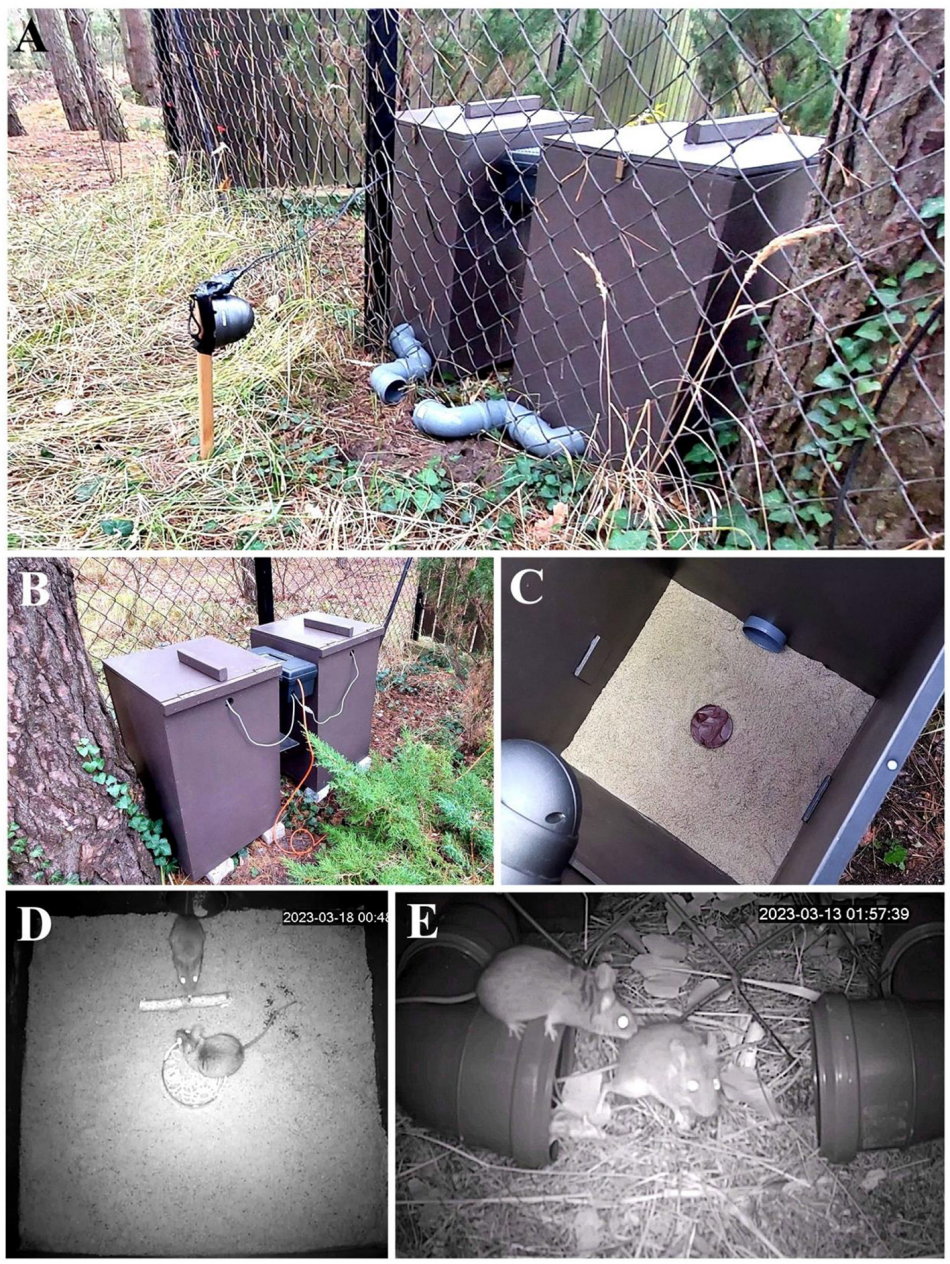
Field FEP in natural conditions demonstrating an assay that eliminates all animal handling. Laboratory-style boxes used for a study on free-ranging rodents (Apodemus mice; *A. agrarius* and *A. flavicollis*). **(A, B)** Two wooden chambers deployed near mice's habitat. **(C)** The inside of the experimental chamber with floor covered with sand layer and chocolate cream as a bait. **(D)** Video-still showing yellow-necked mice (*A. flavicollis*) during social interaction inside one of the chambers. **(E)** Video still showing yellow-necked mice during social interaction near the entrance pipes.

In addition to being welfare-friendly, FEP in the field offers other advantages. Free-living rodents are assumed to be genetically variable and possess their full faculties. This is in contrast to lab animals, which are inbred, have smaller brains, adrenal glands, and different sized brain structures including the basolateral complex of the amygdala, main olfactory bulb, and accessory olfactory bulb (Koizumi et al., 2018), among others. The differences are exacerbated by albinism which is frequent among laboratory rodents and causes impairment in various senses (e.g., Lockard, 1968; Sachs, 1996; Prusky et al., 2002). Additionally, such studies require limited (or no) handing, an unnatural stressor (Sensini et al., 2020) that can influence outcomes. This could be especially relevant to researchers that prefer to pre-identify subjects prior to testing. Additionally, studying animals in their natural environment allows more accurate study of environmental contexts that are missing in standardized laboratory trials. Context is essential because decision rules are often context-specific (e.g., Pinho et al., 2019; Heissenberger et al., 2020). Contexts, such as the availability of conspecifics, competitors, shelter and predators are expected to modify a variety of behaviors of interest. This can be especially important in fear and anxiety studies (Orrock et al., 2004; Orrock and Danielson, 2009; Parsons et al., 2018). Indeed, laboratory and field trial outcomes in olfactory-based research often differ (Apfelbach et al., 2005), and many of these differences can be explained by variable contexts (Parsons et al., 2018; Fendt et al., 2020; Stryjek et al., 2021b). The most parsimonious approach to improve welfare outcomes and increase experimental contexts may be to move laboratory-style chambers into the field (Figure 2; Modlinska and Stryjek, 2016; Stryjek et al., 2018; Bedoya-Pérez et al., 2021; Parsons et al., 2023). A wide range of possible experimental topics are discussed in Stryjek et al. (2021a) and these include studies of novelty, cognition, problem-solving, sensory acuity, behavioral responses to stress (stress resilience), and social behavior.

Millions of rodents are bred and sold to research laboratories each year. By testing wild animals in the wild, fewer rodents have to be bred and there is no need to kill free-living animals. Testing animals in their natural environment may help address the “crisis” of translational medicine as reported by Oppenheim (2019), where he argued that findings from the laboratory are not reliable predictors of clinical outcomes. Finally, studying animals in the wild may be a pathway to identify promising new model system that could be brought back into the laboratory, where they could be more systematically studied using a FEP.

### Laboratory

**Figure 3:**
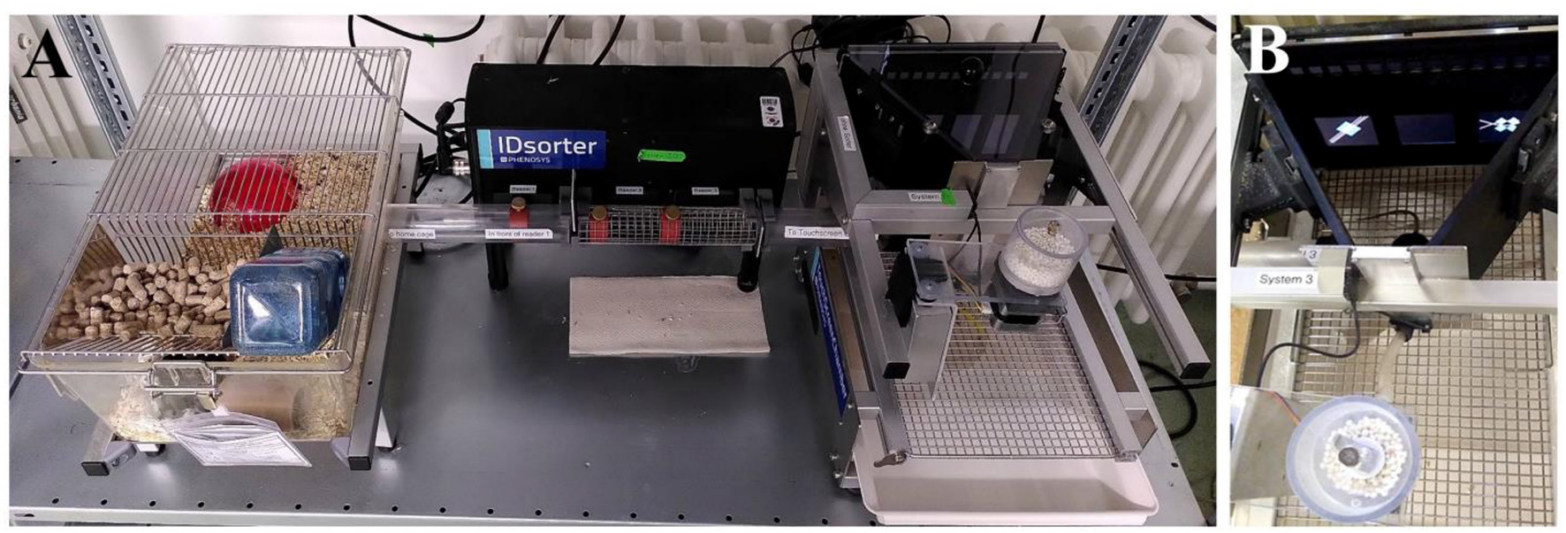
**(A)** Homecage (left), sorter system (middle), and touchscreen box (right). **(B)** Touchscreen box with pictures of a compound discrimination task on the screen (part of the attentional set shifting task measuring cognitive flexibility).

FEP assays when conducted in the laboratory are also welfare-friendly, while the advantages for improving research outcomes are not as straight forward as those in the field. Wild animal studies can be important model systems and by bringing them into laboratory trials they can increase genetic variability and help us better understand natural behavioral variation (Stryjek et al., 2013; Dolivo and Taborsky, 2015; Kiyokawa et al., 2017; Koizumi et al., 2018; Schneeberger et al., 2020). However, animals are still captive. Additionally, animals in traditional paradigms are forced to explore/take part in the study, which elevates stress and distorts the result and can even eliminate behaviors under study. Yet, research in recent decades explored how the FEP could be applied in the lab. Laboratory FEP setups usually consist of a home cage and a testing device, e.g., operant walls (Kiryk et al., 2020), a touch screen box (Rivalan et al., 2017) or mazes such as the radial arm maze (Mei et al., 2020; Kohler et al., 2022). Figure 3 shows an example where the two are connected by a so-called sorter that recognizes the RFID-chipped mouse, ensures that only one animal enters the test device alone, and then starts individual tests in the test device via a connected computer. Mice or rats explore such a setup without food deprivation once they have access to it, quickly become accustomed to the set up, and learn within a few days that they are rewarded (e.g., with sucrose solution or pellets) for solving tasks in the test device. Such a FEP setup can theoretically run 24/7, and the animals visit the test device during both their active and passive phases, making more visits during the active period but surprisingly performing very similar in both phases. Compared to the classical procedure, where animals are manually placed in the boxes for training, the complete training procedure in such an application is several times faster. These studies show that the FEP is useful in field and laboratory conditions.

## Conclusions

There is increasing desire from researchers (Jirkof et al., 2019; Buckland and Nattrass, 2020; d'Isa and Gerlai, 2023), animal welfare advocates, and the public to shift our attitudes about rodents used in experimental research. Indeed, a contingent of researchers and advocates are calling for the replacement of laboratory animals altogether (Gruber and Hartung, 2004; Langley et al., 2007). This has all been happening while traditional rodent research has been under scrutiny (Oppenheim, 2019; Voelkl et al., 2020, 2021) because laboratory animals lack genetic diversity, and because experimental laboratory situations are not similar enough to the “real world” to justify suffering if studies produce questionable results (Manjili, 2013; Drucker, 2016; Matusz et al., 2019; Oppenheim, 2019; Fendt et al., 2020). We recognize however, that laboratory animals are our only means for success in some areas of biomedicine. So, our position is not so strong that we recommend replacing animals altogether, and we recognize that not all research questions can be adequately addressed by the FEP. They do however offer, for some researchers, an intermediate, transitional step, whereby study protocols are explicitly designed to optimize animal welfare and to produce interpretable findings. Ultimately, the FEP, whether the designs we have highlighted, or new designs built to address new questions, can dramatically improve the welfare of rodents, while, when used in the wild, can reduce the number of animals bred and euthanized.

**Figure 4:**
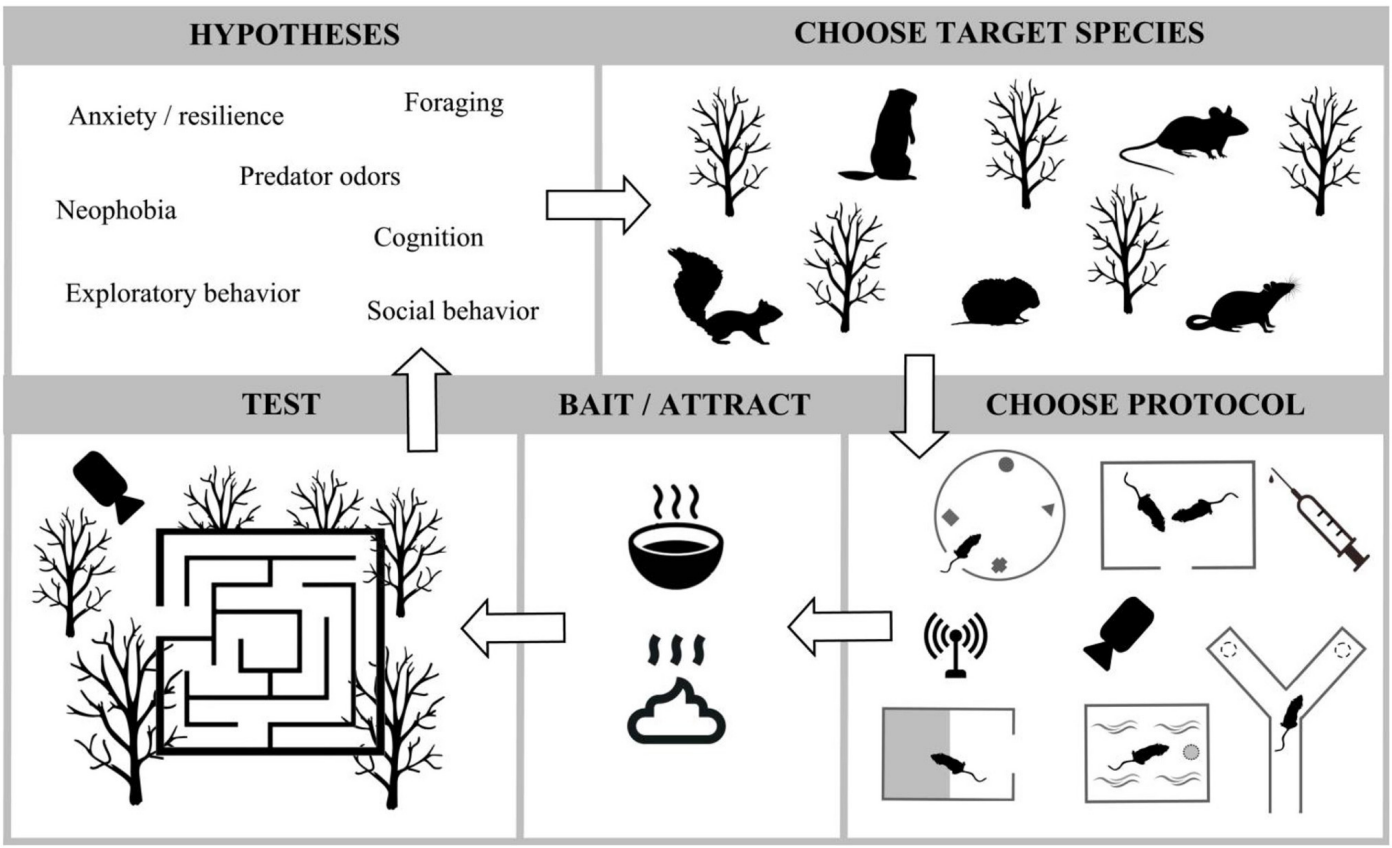
Infographic relating a few generalized types of hypotheses that can be addressed in a range of FEP assays in either the laboratory or the field. As more people work within this paradigm, more types of research questions will be addressed.

The most important misconception we have addressed relates to a common concern about adapting new practices relates to the false assumption that research outcomes will be compromised. FEP in the field may improve research outcomes because they incorporate genetic diversity, minimize animal handling, and take place in a natural environment where many contexts can be isolated or understood in concert with one another. This would satisfy animal welfare concerns and at the same time, address issues about translatability of findings (Drucker, 2016; Oppenheim, 2019). Laboratory FEP may be designed to improve outcomes and welfare in three ways: (1) by increasing heterozygosity when wild animals are brought into controlled settings and allowed to freely enter the designed apparatus; (2) when naturalistic contexts such as availability of conspecifics and shelter are incorporated into laboratory FEP settings; and (3) by minimizing animal handling, we decrease animal stress which is known to cause data distortion (Bailey, 2018). In short, improved welfare also increases data quality. While these types of assays have great potential to improve welfare and for more translatable outcomes, we would be remiss if we did not acknowledge potential shortfalls to be considered during the design and implementation. First, when used in the field where predators are nearby, we recommend deployment of video cameras to look for potential negative impacts on the subjects. In the laboratory, and when using wild-caught animals, care should be taken while catching them and acclimating them; some species may be unsuitable for captive living (Stryjek, 2010; Stryjek et al., 2021b). We hope in the next 10 years, that many variations of the FEP are created to continue addressing our most pressing research questions (for an exhaustive list see Stryjek et al., 2021a, and also Figure 4) in neuroscience, ethology and clinical medicine.

## Author contributions

This invited opinion came about through extended discussions between MP, RS, MF, YK, PB, and DB. MP, RS, MF, YK, PB, and DB wrote and edited the draft. All authors contributed to the article and approved the submitted version.
